# Pneumococcal purpura fulminans in asplenic or hyposplenic patients: a French multicenter exposed-unexposed retrospective cohort study

**DOI:** 10.1186/s13054-020-2769-y

**Published:** 2020-02-26

**Authors:** Damien Contou, Rémi Coudroy, Gwenhaël Colin, Jean-Marc Tadié, Martin Cour, Romain Sonneville, Armand Mekontso Dessap, Nicolas de Prost, for the HOPEFUL Study Group, Laurent Argaud, François Barbier, Amélie Bazire, Gaëtan Béduneau, Frédéric Bellec, Pascal Beuret, Pascal Blanc, Cédric Bruel, Christian Brun-Buisson, Gwenhaël Colin, Delphine Colling, Alexandre Conia, Rémi Coudroy, Martin Cour, Damien Contou, Fabrice Daviaud, Vincent Das, Jean Dellamonica, Nadège Demars, Stephan Ehrmann, Arnaud Galbois, Elodie Gelisse, Julien Grouille, Laurent Guérin, Emmanuel Guérot, Samir Jaber, Caroline Jannière, Sébastien Jochmans, Mathieu Jozwiak, Pierre Kalfon, Antoine Kimmoun, Alexandre Lautrette, Jérémie Lemarié, Charlène Le Moal, Christophe Lenclud, Nicolas Lerolle, Olivier Leroy, Antoine Marchalot, Bruno Mégarbane, Armand Mekontso Dessap, Etienne de Montmollin, Frédéric Pène, Claire Pichereau, Gaëtan Plantefève, Sébastien Préau, Gabriel Preda, Nicolas de Prost, Jean-Pierre Quenot, Sylvie Ricome, Damien Roux, Bertrand Sauneuf, Matthieu Schmidt, Guillaume Schnell, Romain Sonneville, Jean-Marc Tadié, Yacine Tandjaoui, Martial Tchir, Nicolas Terzi, Xavier Valette, Lara Zafrani, Benjamin Zuber

**Affiliations:** 1 Service de réanimation polyvalente, Centre Hospitalier Victor Dupouy, 69, rue du Lieutenant-Colonel Prud’hon, 95100 Argenteuil, France; 2 Groupe de recherche clinique CARMAS, Université Paris Est Créteil, Créteil, France; 3 Service de réanimation médicale, Centre Hospitalier Universitaire de Poitiers, 2 Rue de la Milétrie, 86021 Poitiers, France; 4 INSERM CIC 1402, ALIVE Group, Université de Poitiers, Poitiers, France; 5 Service de réanimation médico-chirurgicale, Centre hospitalier départemental de Vendée, Boulevard Stéphane Moreau, 85925 La Roche-sur-Yon, France; 6 Service de réanimation médicale, Centre Hospitalier Universitaire de Rennes, 2 rue Henri le Guilloux, 35033 Rennes, France; 7 Service de réanimation médicale, Hospices Civils de Lyon, Groupement Hospitalier Edouard Herriot, 3 quai des Célestins, 69002 Lyon, France; 8 Service de réanimation médicale, Hôpital Bichat-Claude Bernard, Assistance Publique-Hôpitaux de Paris, 46, rue Henri Huchard, 75877 Paris Cedex 18, France; 9 Service de réanimation médicale, Hôpital Henri Mondor, Assistance Publique-Hôpitaux de Paris, 51 Avenue du Maréchal de Lattre de Tassigny, 94000 Créteil, France

**Keywords:** Purpura fulminans, Asplenia, *Streptococcus pneumoniae*, Overwhelming post-splenectomy infection, Sepsis

## Abstract

**Background:**

Pneumococcal infections remain the main cause of overwhelming post-splenectomy infections, and purpura fulminans may develop in almost 20% of patients with overwhelming post-splenectomy infection. We aimed at describing the impact of asplenia/hyposplenia on the clinical features and the outcomes of adult patients admitted to the intensive care unit (ICU) for pneumococcal purpura fulminans.

**Methods:**

A 17-year national multicenter retrospective cohort study included adult patients admitted to 55 French ICUs for an infectious purpura fulminans from 2000 to 2016. Patients with pneumococcal purpura fulminans were analyzed according to the absence or presence of asplenia/hyposplenia.

**Results:**

Among the 306 patients admitted to the ICU for purpura fulminans, 67 (22%) had a pneumococcal purpura fulminans, of whom 34 (51%) had asplenia (*n* = 29/34, 85%) or hyposplenia (*n* = 5/34, 15%) and 33 (49%) had eusplenia. The prevalence of pneumococcal purpura fulminans was seven times higher in asplenic/hyposplenic patients compared to eusplenic patients with purpura fulminans (*n* = 34/39, 87% vs. *n* = 33/267, 12%; *p* < 0.001). The median time interval between the occurrence of asplenia/hyposplenia and ICU admission was 20 [9–32] years. Pneumococcal vaccine coverage was 35% in asplenic/hyposplenic patients. Purpura was more frequently reported before ICU admission in asplenic/hyposplenic patients (*n* = 25/34, 73% vs. *n* = 13/33, 39%; *p* = 0.01). The rate of bacteremia did not differ between asplenic/hyposplenic and eusplenic patients (*n* = 31/34, 91% vs *n* = 27/33, 82%; *p* = 0.261). SAPS II (60 ± 14 vs. 60 ± 18; *p* = 0.244) and SOFA (13 [1–5] vs. 14 [1–4, 6]; *p* = 0.48) scores did not differ between asplenic/hyposplenic and eusplenic patients. There were no significant differences between asplenic/hyposplenic and eusplenic patients regarding the rate of limb amputation (*n* = 9/34, 26% vs. 15/33, 45%; *p* = 0.11) and hospital mortality (*n* = 20/34, 59% vs. *n* = 15/33, 45%; *p* = 0.27).

**Conclusions:**

Half of pneumococcal purpura fulminans episodes occurred in asplenic or hyposplenic patients. Pneumococcal vaccine coverage was reported in one third of asplenic/hyposplenic patients. Half of pneumococcal purpura fulminans episodes occurred more than 20 years after splenectomy. Outcomes of pneumococcal purpura fulminans did not show significant differences between patients with or without asplenia or hyposplenia, although the small number of patients included limited our power to detect potential differences between groups.

## Introduction

Approximatively 9000 surgical splenectomies are performed each year in France [1], and the total number of French asplenic or hyposplenic persons is currently estimated to be between 250,000 and 500,000 [1]. Asplenic patients are well-known to be at risk of post-splenectomy infections, mostly caused by *Streptococcus pneumoniae* [2–4]. Such infections may be characterized by a sudden onset and a fulminant course, leading to the so-called overwhelming post-splenectomy infection [5]. A recent multicenter prospective study conducted in 173 German intensive care units (ICU) revealed that purpura fulminans, a rare cause of septic shock carrying high mortality and morbidity [6, 7], may develop in almost 20% of patients with overwhelming post-splenectomy infection [2]. Furthermore, 22 to 32% of infectious purpura fulminans are due to *Streptococcus pneumoniae* [6, 8]. However, data on asplenic or hyposplenic patients with pneumococcal purpura fulminans are scarce, and individual vaccination status is rarely assessed. Moreover, there are only few studies comparing the clinical presentation and the outcome of patients with or without asplenia/hyposplenia admitted to the ICU for an overwhelming sepsis [2].

We aimed to describe the clinical features and outcomes of adult patients admitted to the ICU for a pneumococcal purpura fulminans, according to the absence or presence of asplenia/hyposplenia.

## Methods and patients

We performed an ancillary analysis of a 17-year national multicenter retrospective cohort study including adult patients (≥ 18 years old) admitted to 55 French ICUs for an infectious purpura fulminans from 2000 to 2016. Methods and patients have been previously described [6]. An infectious purpura fulminans was defined by the association of a sudden and extensive purpura, evidence or high clinical suspicion of an infection, whatever its causative microorganism, together with acute circulatory failure needing vasopressor support. Patients with a noninfectious purpura and those with purpura in a context of infectious endocarditis were not included in the study. The investigator of each participating center was responsible for the identification of the patients, either from the hospital medical reports, using the function “research the files in which the word purpura fulminans occurs” of Microsoft Windows®, or through a search using the following International Classification of Diseases (10th Revision) codes: D65 (Disseminated intravascular coagulation), A39 (Meningococcal infection), A40 (Streptococcal sepsis), D65 (Disseminated intravascular coagulation), D69 (Purpura and other hemorrhagic conditions), and G00 (Bacterial meningitis). The hospital discharge reports of all identified patients were anonymized and then electronically or conventionally mailed to the main investigator (DC). Clinical charts were reviewed in order to check the inclusion criteria.

Only patients with pneumococcal (blood or cerebrospinal fluid cultures positive for *Streptococcus pneumoniae*, or positive pneumococcal urinary antigen testing) purpura fulminans were included in the present study. A patient was categorized as asplenic or hyposplenic when the medical records or the imaging performed during the ICU stay revealed the absence of spleen. Counting of Howell-Jolly bodies in peripheral blood smears was not routinely performed neither was the counting of pitted erythrocytes by phase-interference microscopy [5]. Asplenic/hyposplenic patients were compared to eusplenic patients (defined as patients without asplenia or hyposplenia) admitted to the ICU for a pneumococcal purpura fulminans.

### Collection of data

Upon ICU admission and during ICU stay, data pertaining to demographics, comorbidities, clinical examinations, laboratory findings, microbiological investigations, and therapeutic management were collected. Simplified Acute Physiology Score II (SAPS II) [9] and Sequential Organ Failure Assessment (SOFA) [10] scores were computed using the worst values recorded within the first 24 h of admission. Missing data were retrieved from queries to the investigators. In asplenic/hyposplenic patients, the following data were retrieved from the ICU discharge reports: cause of asplenia (congenital or post-splenectomy in case of trauma, hematological diseases or cancer) or hyposplenia (splenic irradiation, sickle cell disease), time interval between asplenia/hyposplenia and ICU admission, presence or absence of antibiotic prophylaxis, and pneumococcal vaccination status.

### Statistical analysis

Data were compared between asplenic/hyposplenic and eusplenic patients. Continuous variables were reported as median [25th–75th interquartile range] (IQR) or mean ± standard deviation (SD) and compared between groups using the Student *t* test or Mann-Whitney test, as appropriate. Categorical variables were reported as numbers and percentages (95% confidence interval, CI) and compared using *χ*^2^ test or Fisher’s exact test, as appropriate. All statistical analyses were conducted using the SPSS Base 21.0 statistical software package (SPSS Inc., Chicago, IL.). A *p* value < 0.05 was considered statistically significant.

### Ethical considerations

This observational, non-interventional analysis of medical records was approved by the Institutional Review Board of the French Society of Intensive Care (FICS) in March 2016. As per French law, no informed consent was required for this type of study.

## Results

### Description of asplenic or hyposplenic patients with pneumococcal purpura fulminans

**Fig. 1:**
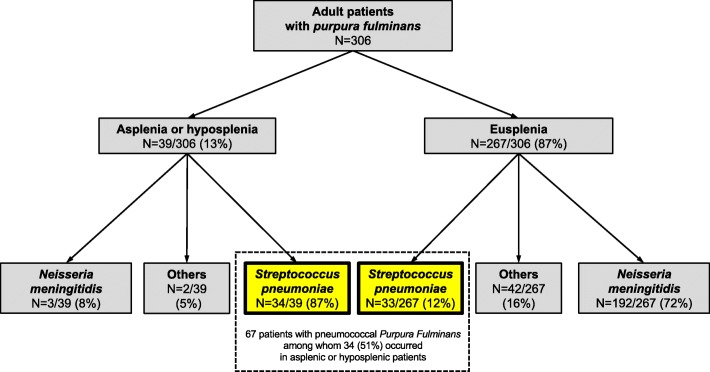
Flow chart of adult patients with pneumococcal *purpura fulminans* with asplenia/hyposplenia (*n* = 34) or eusplenia (*n* = 33)

Among the 306 patients admitted to the ICU for purpura fulminans, 67 (22%) had a pneumococcal purpura fulminans, of whom 34 (51%) had asplenia (*n* = 29/34, 85%) (Table 1) or hyposplenia (*n* = 5/34, 15%) and 33 (49%) had eusplenia. Age, gender, and ICU severity scores did not differ between asplenic/hyposplenic and eusplenic patients (Table 2). The prevalence of pneumococcal purpura fulminans was seven times higher in asplenic or hyposplenic patients compared to eusplenic patients with purpura fulminans (*n* = 34/39, 87% vs. *n* = 33/267, 12%; *p* < 0.001) (Fig. 1). The main causes of asplenia were post-splenectomy (*n* = 26/29, 90%) and congenital (*n* = 3/29, 10%, two cases being diagnosed during ICU stay) (Table 1**)**. Five patients had hyposplenia related to a splenic irradiation for lymphoma. The median time interval between asplenia/hyposplenia and ICU admission was 20 [9–32] years, with only 2/34 (6%) patients having pneumococcal purpura fulminans within the 2 years following splenectomy/hyposplenia (Table 1). Only one patient (*n* = 1/32, 3%) (2 missing data) had received long-term antibiotic prophylaxis (time interval between splenectomy and ICU admission < 1 year). Pneumococcal vaccination had been performed in 11/31 (35%) patients (3 missing data) (Table 1). Among the 67 patients with pneumococcal purpura fulminans, 58 (87%) had positive blood cultures, 25 (37%) had a positive pneumococcal urinary antigen testing, and 11 of the 29 (38%) patients who had lumbar puncture had a positive CSF culture (Table 2).

**Table 1:** Description of the 34 asplenic or hyposplenic patients with pneumococcal purpura fulminans

	Hyposplenic or asplenic patients, *N* = 34
Asplenia	29/34 (85%)
Hyposplenia	5/34 (15%)
Cause of asplenia	
Congenital	3/29 (10%)
Post-splenectomy	26/29 (90%)
Trauma	11/26 (42%)
Immune thrombocytopenic purpura	6/26 (23%)
Pancreatic cancer	3/26 (12%)
Left diaphragmatic hernia	1/26 (4%)
Lymphoma	1/26 (4%)
Myelodysplasia	1/26 (4%)
Benign splenomegaly	1/26 (4%)
Unknown	1/26 (4%)
Cause of hyposplenia	
Splenic irradiation	5/5 (100%)
Sickle cell disease	0/5 (0%)
Time interval between asplenia/hyposplenia and ICU admission, and pneumococcal vaccination coverage	
Time interval between asplenia/hyposplenia and ICU admission (years)	20 [9–32]
Time interval between asplenia/hyposplenia and ICU admission ≤ 2 years	2/34 (6%)
Time interval between asplenia/hyposplenia and ICU admission (years)*	19 [9–30]
Time interval between asplenia/hyposplenia and ICU admission ≤ 2 years*	2/31 (6%)
Long-term antibiotic prophylaxis	1/32 (3%)
Pneumococcal vaccination	11/31 (35%)

*After exclusion of the three patients with congenital asplenia

**Table 2:** Comparison between patients with pneumococcal purpura fulminans having asplenia/hyposplenia (*n* = 34) or eusplenia (*n* = 33)

	Hypo/asplenia, *N* = 34	Eusplenia, *N* = 33	*p*
Age, gender, and ICU scores			
Age, years	48.9 ± 14.4	49.2 ± 14.1	0.83
Male gender	22 (65)	15 (45)	0.11
SAPS II	60 ± 14	60 ± 18	0.24
SOFA	13 [12–16]	14 [10–15]	0.48
Comorbidities			
No coexisting comorbid conditions^a^	24 (71)	20 (61)	0.39
Chronic respiratory disease	1 (3)	0 (0)	0.32
Chronic heart failure	1 (3)	0 (0)	0.32
Chronic kidney disease	0 (0)	0 (0)	–
Cirrhosis	0 (0)	1 (3)	0.31
Alcohol use	2 (6)	7 (21)	0.07
Recent malignant hemopathy	1 (3)	1 (3)	0.98
Recent cancer	0 (0)	0 (0)	–
Diabetes mellitus	3 (9)	1 (3)	0.32
HIV infection	1 (3)	2 (6)	0.98
Obesity	0 (0)	1 (3)	0.31
Cerebrovascular disease	0 (0)	1 (3)	0.31
Immunosuppressive therapy	2 (6)	2 (6)	0.98
Prior to ICU admission			
Fever	29 (85)	31 (94)	0.25
Headache	12 (35)	14 (42)	0.55
Digestive signs	25 (73)	16 (48)	0.04
NSAIDs consumption	5 (56)	4 (44)	0.70
General practitioner visit in the prior 48 h	10 (29)	7 (21)	0.44
Purpura before ICU admission	25 (73)	13 (39)	0.01
Antibiotic therapy before ICU-admission	25 (73)	21 (64)	0.38
Dysphagia/odynophagia	2 (6)	1 (3)	0.57
Myalgia	8 (23)	4 (12)	0.22
Arthralgia	4 (6)	1 (3)	0.17
Lower limb pain	13 (38)	4 (12)	0.01
At ICU admission			
Coma Glasgow score	14 [13–15]	15 [13–15]	0.99
Temperature, °C	38.3 [37.4–39.0]	38.6 [38.0–40.0]	0.11
Neck stiffness	4 (12)	2 (6)	0.41
Lower limb hyperalgesia	14 (41)	7 (21)	0.08
Biological data at ICU admission			
Leukocytes count, 10^3^ mm^−3^	10.9 [6.0–14.9]	9.7 [1.6–21.0]	0.34
Platelets count, 10^3^ mm^−3^	32 [19–49]	36 [19–67]	0.74
C-reactive protein, mg/L	176 ± 86	276 ± 155	0.01
Procalcitonin, ng/mL	113.5 ± 66.5	96.2 ± 59.0	0.73
Serum urea, mmol/L	13.0 [11.1–19.0]	12.0 [8.6–15.0]	0.14
Serum creatinine, μmoL/L	257 [195–310]	211 [174–303]	0.22
Prothrombin time, %	24 [14–35]	33 [18–48]	0.12
Fibrinogen, g/L	0.6 [0.4–1.7]	2.0 [0.9–3.4]	0.04
Arterial lactate, mmol/L	8.0 [4.9–10.0]	7.4 [5.6–11.9]	0.74
Troponin, mg/L	0.2 [0.1–2.6]	1.0 [0.1–35.7]	0.55
Creatine kinase, IU/L	677 [210–7825]	812 [377–4355]	0.75
Microbiological data			
Bacteraemia	31 (91)	27 (82)	0.26
Lumbar puncture performed	13 (38)	16 (48)	0.40
CSF white blood cell counts, 10^3^ mm^−3^	3 [1–5]	7 [3–277]	0.06
Meningitis^b^	2 (17)	6 (43)	0.15
Positive culture	6 (46)	5 (31)	0.41

*Abbreviations*: *CSF* cerebrospinal fluid, *ICU* intensive care unit, *NSAID* nonsteroidal anti-inflammatory drugs, *SAPS* Simplified Acute Physiology Score, *SOFA* Sequential Organ Failure Assessment; ^a^other than asplenia/hyposplenia; ^b^defined as ten or more cell/mm^3^ in cerebrospinal fluid

### Clinical presentation, biological and microbiological features in asplenic/hyposplenic and eusplenic patients with pneumococcal purpura fulminans

Clinical presentation did not significantly differ between asplenic/hyposplenic and eusplenic patients, except for digestive symptoms (*n* = 25/34, 73% vs. *n* = 16/33, 48%; *p* = 0.04) and lower limb pain (*n* = 13/34, 38% vs. *n* = 4/33, 12%; *p* = 0.01), which were more frequently reported in the former than in the latter. Purpura was also more frequently reported before ICU admission in asplenic/hyposplenic patients than in their counterparts (*n* = 25/34, 73% vs. *n* = 13/33, 39%; *p* = 0.01) (Table 2). Biological and microbiological data did not differ between asplenic/hyposplenic and eusplenic patients. The rate of bacteremia was > 80% but did not significantly differ between hyposplenic/asplenic and eusplenic patients (*n* = 31/34, 91% vs. *n* = 27/33, 82%; *p* = 0.26). There was no significant difference between groups regarding antibiotic therapy before ICU admission (*n* = 25/35, 73% vs. *n* = 21/33, 64%, *p* = 0.38), and all patients received a beta-lactam antibiotic in the ICU. Details regarding comorbidities, ICU scores, clinical presentation, and biological and microbiological data were provided in Table 2.

### Management, organ supports and outcomes in asplenic/hyposplenic and eusplenic patients with pneumococcal purpura fulminans

**Fig. 2:**
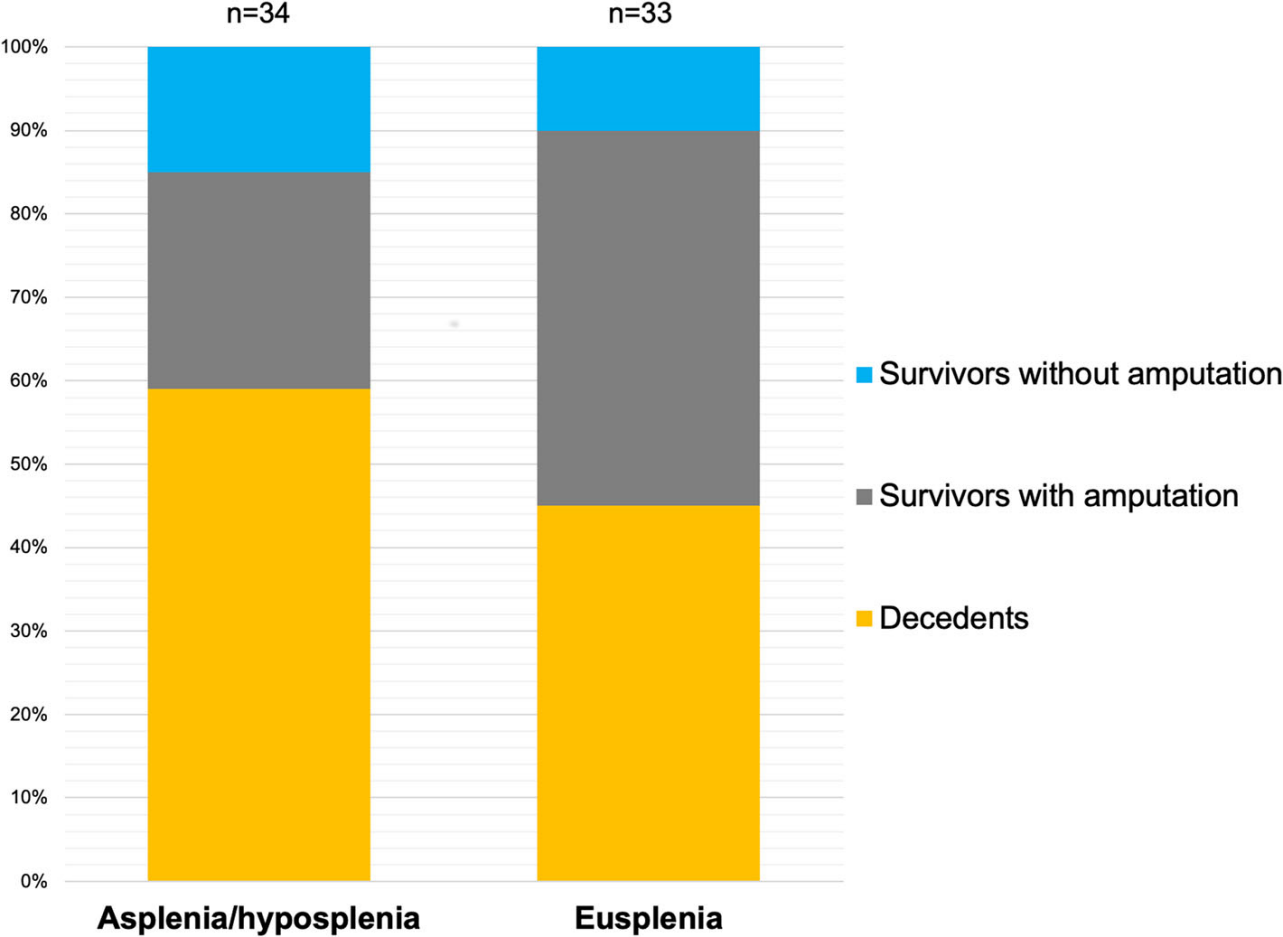
Comparison of outcomes between asplenic or hyposplenic (*n* = 34) and eusplenic (*n* = 33) patients with pneumococcal *purpura fulminans*

The rates of invasive mechanical ventilation, renal replacement therapy, systolic myocardial dysfunction, and platelet or plasma transfusions did not differ between asplenic/hyposplenic and eusplenic patients (Table 3), nor did the rate of limb amputation (*n* = 9/34, 26% vs. 15/33, 45%; *p* = 0.11) or hospital mortality (*n* = 20/34, 59% vs. *n* = 15/33, 45%; *p* = 0.27) (Fig. 2). Among asplenic/hyposplenic patients, hospital mortality did not differ between vaccinated and unvaccinated patients (*n* = 4/9, 44% vs. *n* = 14/22, 63%; *p* = 0.60). Outcome did not differ between hyposplenic and asplenic patients regarding limb amputations (*n* = 1/5, 20% vs. 8/29, 28%; *p* = 0.78) and hospital mortality (*n* = 3/5, 60% vs. 17/29, 59%; *p* = 0.98). More details regarding management, organ supports, and outcomes are provided in Table 3.

**Table 3:** Management, organ supports and outcomes in patients with pneumococcal purpura fulminans having asplenia/hyposplenia (*n* = 34) or eusplenia (*n* = 33)

	Hypo/asplenia, *N* = 34	Eusplenia, *N* = 33	*p*
Invasive mechanical ventilation	32 (94)	33 (100)	0.16
Duration of mechanical ventilation, days	11 [2–33]	9 [4–17]	0.56
ARDS	20 (59)	14 (42)	0.18
Renal replacement therapy	22 (65)	23 (70)	0.66
Lowest LVEF	30 [29–50]	26 [11–53]	0.11
Systolic myocardial dysfunction (LVEF< 45%)	18 (72)	17 (71)	0.93
Inotropic agent	17 (61)	18 (62)	0.92
ECMO	0 (0)	6 (18)	0.01
Steroids for septic shock or meningitis	25 (73)	20 (61)	0.26
Activated protein C	6 (18)	3 (9)	0.31
Platelets transfusion	22 (65)	24 (73)	0.48
Plasma transfusion	22 (65)	22 (67)	0.87
Duration of vasopressor therapy, days	5 [2–13]	5 [3–7]	0.41
Duration of ICU stay, days	9 [2–45]	15 [4–32]	0.96
Limb amputation, %	9 (26)	15 (45)	0.11
In-hospital mortality, %	20 (59)	15 (45)	0.27

Abbreviations: *ARDS* acute respiratory distress syndrome, *LVEF* left ventricle ejection fraction, *ECMO* extra-corporeal membrane oxygenation, *ICU* intensive care unit

## Discussion

We report on the first multicenter cohort of adult patients admitted to the ICU for a pneumococcal purpura fulminans. The main results are as follows: (1) half of pneumococcal purpura fulminans occurred in asplenic or hyposplenic patients, (2) pneumococcal vaccination coverage was only 29% in adult asplenic/hyposplenic patients with pneumococcal purpura fulminans, (3) the time interval between asplenia/hyposplenia and pneumococcal purpura fulminans was 20 years, and (4) outcomes did not differ between patients with or without asplenia/hyposplenia. These results must be interpreted with caution due to the relatively small number of patients included, which limited our ability to show significant statistical differences between groups. Our study is, to the best of our knowledge, the first one to compare clinical features and outcomes of patients with pneumococcal purpura fulminans according to the presence/absence of a spleen, rendering comparisons with other studies difficult.

The median time interval between asplenia/hyposplenia and pneumococcal purpura fulminans observed in our study was 20 years, with only very few patients with pneumococcal purpura fulminans occurring within the 2 years following splenectomy or hyposplenia. This time interval is longer than the 5.75 years time interval observed in a recent prospective multicenter German study on overwhelming post-splenectomy infection from every cause and pathogen [2]. It is commonly accepted that the greatest risk of overwhelming post-splenectomy infection is maximal within the first 2 years after splenectomy [11–13]. However, most of these studies [11–13] included immediate post-operative infections and had a limited follow-up duration, potentially underestimating the risk of late overwhelming post-splenectomy infection. The time interval observed in our cohort highlights that the risk of overwhelming post-splenectomy infection remains lifelong [3, 4, 14, 15].

In the present study, only 35% of the patients with asplenia or hyposplenia had received a pneumococcal vaccination despite current recommendations of the Centers for Disease Control and Prevention [16]. However, it is worth noticing that 2/34 (6%) patients were not aware of their asplenic status since asplenia was diagnosed in the ICU and revealed by the pneumococcal purpura fulminans episode. Moreover, given the wide study period (17 years), recommendations about pneumococcal vaccination may have varied over time. The vaccination rate observed in our cohort is consistent with the 31% rate reported by Waghorn et al. in a series of 77 patients with overwhelming post-splenectomy infection [3]. In a recent multicenter German cohort study including 52 patients with overwhelming post-splenectomy infection, only 11 (21%) had received a pneumococcal vaccination within the past 5 years. In the global population of splenectomized patients without infection, the coverage of pneumococcal vaccination rates is as high as 80% [17, 18]. Our finding of one third of the asplenic/hyposplenic patients with pneumococcal purpura fulminans having received a pneumococcal vaccination emphasizes that pneumococcal vaccination may reduce but does not abolish the risk of overwhelming post-splenectomy infection in splenectomized patients.

Our study showed similar severity levels and outcomes between patients with and without asplenia/hyposplenia. This observation is in line with a prospective multicenter German cohort study reporting a similar ICU, 7-day or 28-day mortality between patients with overwhelming post-splenectomy infection (from every cause and pathogen) and matched patients without asplenia or hyposplenia [2]. Similarly, a large prospective Canadian cohort study including 2435 patients with invasive pneumococcal disease over a 15-year period reported on similar in-hospital mortality rates between asplenic/hyposplenic and eusplenic non-ICU patients [19]. This observation underlines the fact that asplenia is a risk factor for severe pneumococcal sepsis but seems not to worsen the course of a declared sepsis, pointing out that our efforts must focus on preventing the disease. In this regard, a retrospective Australian before-after study recently showed that including patients in a registry of asplenic patients (with educational kit containing information sheet, alert cards regarding the history of splenectomy, educational DVD, personalized vaccine report, information regarding antibiotic prophylaxis, emergency plan in case of fever, newsletter to their general practitioners with updates regarding recommended vaccinations and booster doses) was associated with a significant reduction in the incidence of infections caused by encapsulated bacteria after splenectomy [4].

### Limitations

Our study certainly suffers from several limitations. This was a retrospective study with inherently associated bias, as well as missing data and possible associated errors in data abstraction. However, due to the extreme rarity of pneumococcal purpura fulminans, a prospective study would be hardly feasible. A previous prospective multicenter study on overwhelming post-splenectomy infection in 173 German ICUs prematurely ended due to slow recruitment [2]. Abdominal CT scan and detection of Howell-Jolly bodies in peripheral blood smears or counting of pitted erythrocytes by phase-interference microscopy was not routinely performed. Therefore, we cannot exclude that patients included in the eusplenic group had an unknown asplenia potentially related to congenital asplenia or other diseases (severe celiac disease, inflammatory bowel disease, Whipple’s disease, amyloidosis, Sjögren’s syndrome, HIV infection, cirrhosis) associated with hyposplenism or splenic atrophy [5, 20]. However, only 15% (*n* = 5/33) of the eusplenic patients had pre-existing diseases potentially associated with hyposplenism (ulcerative colitis *n* = 1, Sjögren’s syndrome *n* = 1, HIV infection *n* = 2, cirrhosis *n* = 1). Because the patients were recruited over a 17-year period in 55 centers, ICU procedures were inevitably heterogeneous. The serotypes of the *Streptococcus pneumoniae* strains involved was not available due to the retrospective nature of the study, precluding any comparison to be made between both groups of patients. Last, the absence of significant outcome differences observed between asplenic/hyposplenic and eusplenic patients may be due to a lack of power related to a relatively small number of included patients.

Our study also has some strengths, including the large number of centers and patients included for a very rare infectious disease [6], the comparison between asplenic and non-asplenic patients, with a paucity of previously published data due to the rarity of overwhelming post-splenectomy infection [2], the collection of pneumococcal vaccination status which is poorly reported, and the fact that the definitions used for inclusion of the patients were well-standardized, rendering the comparison of the two groups relevant.

### Clinical implications

Asplenic or hyposplenic patients admitted to the ICU for a purpura fulminans seem to have a high risk of pneumococcal purpura fulminans. In patients surviving pneumococcal purpura fulminans, unknown congenital asplenia should be searched with abdominal CT-scan. Detecting Howell-Jolly bodies in peripheral blood smear and counting pitted erythrocytes by phase-interference microscopy may also help diagnosing hyposplenia [5]. In such patients, pneumococcal vaccination seems welcomed in order to avoid recurrences of overwhelming post-splenectomy infection. As recommended by the Centers for Disease Control and Prevention 2019 guidelines [16], patients with asplenia or hyposplenia should receive one dose of the 13-valent pneumococcal conjugate vaccine together with one (for persons aged ≥ 64 years) or two doses (for persons aged 19–64 years) of the 23-valent pneumococcal polysaccharide vaccine. Furthermore, continuing education is required to advise patients with a risk of severe infection inherent to asplenia and the need for immediate antibiotic therapy in case of fever, especially since it has been reported that 40 to 84% of splenectomized individuals were not aware of the infectious risks associated with their condition [18, 21]. In adult asplenic/hyposplenic patients, long-term antibiotic prophylaxis [5] is still debated, except in those who survived overwhelming post-splenectomy infection [20], a targeted subgroup of patients in whom such a strategy seems appropriate, although supported by a low evidence.

## Conclusion

Half of pneumococcal purpura fulminans occurred in asplenic or hyposplenic patients, who had a poor vaccination coverage against *Streptococcus pneumoniae*. Half of pneumococcal purpura fulminans episodes occurred more than 20 years after splenectomy. Outcomes seemed not to differ between asplenic/hyposplenic and eusplenic patients.

## Data Availability

The datasets used and/or analyzed during the current study are available from the corresponding author on reasonable request.

## Abbreviation

ICU: Intensive care unit
